# Novel type II toxin-antitoxin systems with VapD-like proteins

**DOI:** 10.1128/mbio.00003-25

**Published:** 2025-03-07

**Authors:** Konstantin Gilep, Dmitry Bikmetov, Aleksandr Popov, Anastasiia Rusanova, Shunsuke Tagami, Svetlana Dubiley, Konstantin Severinov

**Affiliations:** 1 Center for Precision Genome Editing and Genetic Technologies for Biomedicine Institute of Gene Biology, Russian Academy of Sciences, Moscow, Russia; 2 Institute of Gene Biology, Russian Academy of Sciences, Moscow, Russia; 3 National Research Center “Kurchatov Institute”, Moscow, Russia; 4 RIKEN Center for Biosystems Dynamics Research, Yokohama, Kanagawa, Japan; 5 Graduate School of Medical Life Science, Yokohama City University, Yokohama, Kanagawa, Japan; 6 Koltzov Institute of Developmental Biology, Russian Academy of Sciences, Moscow, Russia; 7 Graduate School of Medicine, Science and Technology Shinshu University, Matsumoto City, Nagano, Japan; 8 International Institute for Sustainability with Knotted Chiral Meta Matter (WPI-SKCM²) Hiroshima University, Higashi-Hiroshima, Hiroshima, Japan; 9 Waksman Institute for Microbiology, Piscataway, New Jersey, USA; University of Pittsburgh, Pittsburgh, Pennsylvania, USA

**Keywords:** toxin-antitoxin systems, VapD, SOS-response, Cas2, evolution

## Abstract

Type II toxin-antitoxin (TA) systems are widespread in prokaryotes. They consist of neighboring genes encoding two small proteins: a toxin that inhibits a critical cellular process and an antitoxin that binds to and neutralizes the toxin. The VapD nuclease and the VapX antitoxin comprise a type II TA system that contributes to the virulence of the human pathogen *Haemophilus influenzae*. We analyzed the diversity and evolution of VapD-like proteins. By examining loci adjacent to genes coding for VapD-like proteins, we identified two novel families of antitoxins, which we named VapY and VapW. VapD toxins cognate to novel antitoxins induce the SOS response when overproduced, suggesting they target cellular processes related to genomic DNA integrity, maintenance, or replication. Though VapY has no sequence similarity to VapX, they share the same SH3 fold characterized by the five anti-parallel β sheets that form a barrel. VapW is a homolog of VapD without conserved catalytic residues required for nuclease activity. The crystal structure of the VapD-VapW complex reveals that VapW lacks the dimerization interface essential for the catalytic activity of VapD but retains the second interaction interface that enables VapD hexamerization. This allows VapW to bind VapD in the same manner that VapD dimers bind to each other in hexamers. Thus, though the VapD catalytic cleft remains accessible in the VapD-VapW complex, VapW may disrupt VapD oligomerization. To our knowledge, VapWD provides a unique example of TA systems evolution when a toxin loses its activity and becomes an antitoxin to itself.

**IMPORTANCE:**

Genes encoding virulence-associated protein D (VapD) homologs are found in many pathogens such as *Helicobacter pylori*, *Haemophilus influenzae*, and *Xylella fastidiosa*. There are many indications that VapD proteins contribute to virulence, even though the exact mechanism is not known. VapD proteins are either encoded by stand-alone genes or form toxin-antitoxin pairs with VapX. We performed a comprehensive census of vapD-like genes and found two new antitoxins, VapW and VapY. The VapW antitoxins are catalytically inactivated variants of VapD, revealing a new evolutionary mechanism for the appearance of toxin-antitoxin pairs.

## INTRODUCTION

Toxin-antitoxin (TA) systems are genetic elements commonly present in prokaryotic plasmids and genomes. These systems were shown to stabilize plasmids (1–4), modulate cellular responses to various stresses, and contribute to phage defense (5–7). Most TA systems are composed of two components—a toxin that impairs an essential process in the cell and a cognate antitoxin that restrains the toxin activity. Both components can be represented by either a protein or an RNA. Based on the nature of components, as well as the mechanism of toxin neutralization, the TA systems are divided into eight types (8) (for a comprehensive review, see references 5, 8, 9).

In type II TA systems, both toxin and antitoxin are small proteins. The antitoxin directly binds the cognate toxin rendering it inactive. Inactivation of a type II toxin by its antitoxin can be achieved through one of the following mechanisms: occlusion of the toxin catalytic site, steric hindrance that impedes target or cofactor accessibility, secondary or tertiary structure disruption (e.g., rearrangement of catalytic residues or inhibition of toxin oligomerization), and various combinations thereof (10). Toxins of the same family can be associated with non-homologous antitoxins and vice versa (11, 12). The evolutionary plasticity of TA systems is further highlighted by the ability to directly convert a toxin to an antitoxin using protein engineering (13). The VapD protein was initially discovered as a component of the virulence-associated genetic region *vapABCD* in sheep pathogen *Dichelobacter nodosus* (14). Later, a VapD homolog was identified in a common human pathogen *Haemophilus influenzae* (15), where it forms a type II TA system VapXD^Hin^ with the VapX antitoxin (16). VapD from *D. nodosus* and VapD HP0315 from *Helicobacter pylori* (VapD^Hpy^) have no associated antitoxins (17). VapD^Hin^ and VapD^Hpy^ *in vitro* demonstrated non-specific RNase activity, predominantly cleaving RNA before purine residues (16, 17), but no *in vivo* targets of these or any other VapD are known. Several studies proposed that VapD proteins contribute to virulence and stress response (18–22)

The VapD proteins have a ferredoxin-like fold (18, 23). The minimal nuclease-active form of VapD^Hpy^ and VapD^Hin^ is a dimer, with each subunit jointly forming a cleft that contains the putative catalytic site with conserved aspartate and serine residues (17, 23). VapX is a monomeric single-domain β-barrel protein. One subunit of VapX binds the VapD dimer and occludes the putative catalytic cleft, thereby preventing the nucleolytic activity (23). Purified VapD^Hin^ exists in the form of a flat “puck-shaped” homohexamer (a trimer of dimers) (23).

VapD proteins are distant homologs of the Cas2 proteins encoded by most CRISPR-Cas adaptive immunity systems (24). Cas2 binds to its partner, Cas1, forming an adaptation complex—a site-specific integrase responsible for the acquisition of new DNA spacers into CRISPR arrays (25). Some Cas2 proteins possess RNase or DNase activity (26–28), which, however, is dispensable for spacer acquisition (25).

In this work, we performed a phylogenetic analysis of VapD-like proteins coupled with the analysis of proteins encoded by associated genes. This approach revealed two new families of type II TA systems that comprise a VapD toxin paired with novel antitoxins, which we named VapY and VapW. The VapW antitoxins are VapD homologs but lack the catalytic residues. Structural characterization of the VapD-VapW complex showed that VapW does not interfere with the putative catalytic site of the VapD dimer. Instead, VapW binds at the interface required for VapD homohexamerization. Tested VapY- and VapW-associated VapD toxins cause SOS response in *Escherichia coli*, suggesting that these toxins target genomic DNA replication and/or maintenance.

## MATERIALS AND METHODS

### Bioinformatic analysis

Six hundred seventeen protein sequences from the UniProt database (29) classified as VapD according to the PIRSF system (30) were collected and clustered using MMseqs2 with 90% identity and 90% coverage cutoffs (31). The resulting non-redundant data set, consisting of 199 proteins, was aligned using MUSCLE with default parameters (32). The multiple sequence alignment (MSA) was used as a query for PSI-BLAST search against the NCBI nr database (33) with profile-inclusion and reporting *E*-value cutoffs of 0.005 and 10, respectively. The search converged after 21 iterations and returned 2,727 unique sequences. For all databases, versions as of February 2022 were used. To construct an outgroup for rooting of the VapD phylogenetic tree, we performed another search with the same query and an *E*-value cutoff of 100 against a set of Cas2 proteins from the InterPro database (families “IPR019199,” “IPR021127,” and “IPR010152”) (34). Sequences selected for the outgroup were labeled, and the results of the two searches were combined. For proteins longer than 125 amino acids, only regions aligned by PSI-BLAST were used for further analysis. Proteins shorter than 47 amino acids were excluded. The resulting data set was clustered using MMseqs2 with 90% identity and 90% coverage thresholds. If a representative sequence appeared to be from the PDB database, it was replaced with a random protein from the corresponding cluster, when possible, to obtain the information about the genome that encodes it. The same procedure was applied for representative sequences labeled as the “outgroup” since the InterPro family “IPR019199” includes VapD-like proteins. Representative sequences from each cluster were aligned using MUSCLE with a gap opening penalty of −10, and the multiple sequence alignment (MSA) was trimmed using Clipkit with defaults. The resulting alignment of 1,182 sequences was then used to build a phylogenetic tree using RAxML version 8.2.12 (35) with a gamma model of rate heterogeneity and LG substitution matrix (36). Four hundred rapid bootstrap replications were performed until convergence according to the autoMRE criterion. Transfer bootstrap expectation values were calculated using BOOSTER (37). The final tree was visualized with the iTOL (38).

For every protein from the tree except for the outgroup, one genomic assembly containing the corresponding gene was downloaded via Entrez (33). If the contig contained only one gene, the representative sequence was replaced by another sequence from the same cluster when possible. FlaGs (39) was used for the clusterization of proteins encoded within eight CDSs upstream or downstream from genes of VapD homologs and their functional annotation with profile HMMs from the Pfam (40) and TIGRFAMs (41) databases. Members of the three families of putative antitoxins obtained from FlaGs were aligned using MUSCLE with default parameters, and profile hmms were built using the program hmmbuild from the HMMER package version 3.3.2 (http://hmmer.org/). To identify VapD protein fusions, portions of VapD-like proteins from the tree that were not aligned during the initial PSI-BLAST search and were longer than 20 amino acids were excised and scanned with three putative antitoxins hmms via hmmscan. Identified VapD homologs and analysis of the adjacent genes are provided in Table S1.

The structural alignment of VapD^Hin^ (PDB ID: 6ZN8, https://www.rcsb.org/structure/6ZN8), *VapD*^Hpy^ (PDB ID: 3UI3, https://www.rcsb.org/structure/3UI3), and both VapD^Cje^ and VapW^Cje^ (this work) was obtained using Matt (42) with default parameters. Improved sequence alignment of VapW and VapD-like proteins was performed via MAFFT (43) using structural alignment as a constraint with a gap opening penalty 5 and visualized using JalView (44). Sequence logos for VapD and VapW multiple alignments were generated using the WebLogo online tool (45).

### Plasmid construction

Synthetic DNA fragments of *vapY*, *vapW*, and *vapD* genes were codon-optimized for *E. coli* and ordered from Evrogen, Russia, or Integrated DNA Technologies, USA (Table S2). Primers used in this study are listed in Table S3. The *vapD* genes were cloned into the pBAD33 vector, and *vapW* or *vapY* was inserted into pASK-IBA43plus. A plasmid producing the D7N VapD^Mur^ mutant was obtained by site-directed mutagenesis using overlap-extension PCR (46). For protein purification, pRSF-Duet1-based plasmids containing *vapD* toxin genes and cognate *vapW* or *vapY* antitoxin genes fused to his-tag or Strep-tag, respectively, were constructed. Detailed protocols of plasmid construction are described in supplemental methods.

### Cell toxicity test

*E. coli* BW25113 was co-transformed with pBAD33- and pASK-based plasmids. Although pBAD33 and pASK plasmids bear different origins of replication, a previous study reported that pBAD33 can induce the loss of ColE1 after co-transformation due to segregation issues (47). Throughout, we employed dual antibiotic selection to ensure the maintenance of both plasmids (pBAD33: chloramphenicol and pASK: ampicillin). Several freshly grown colonies were inoculated in 2× YT media supplemented with chloramphenicol (34 µg/mL) and ampicillin (100 µg/mL). Cultures were grown at 37°C until optical density (OD_600_) of 0.3, at which point cultures were split and inducers (0.1 µg/mL anhydrotetracycline [AtC] and 0.1% [wt/vol] arabinose [ara]) were added. Cultures were further incubated for 1 or 2 h at 37°C, and serial 10-fold dilutions were plated on 2× YT agar plates containing 1.5% agar, 1% glucose, and appropriate antibiotics.

In the first toxicity test setup, cells harbored a pBAD33-based plasmid encoding a VapD toxin and a pASK plasmid encoding either VapW or VapY cognate antitoxins (e.g., pBAD33_VapD-Mur and pASK_VapY-Mur). The following combinations of inducers were tested: (i) AtC only, (ii) ara only, (iii) both ara and AtC, and (iv) no inducers. In the second approach, four cell cultures, each with different combinations of empty or gene-carrying plasmids, were tested: (i) pBAD33 and pASK-IBA43plus vectors, (ii) toxin-harboring pBAD33 with the pASK-IBA43plus vector, (iii) the pBAD33 vector with antitoxin-harboring pASK-IBA43plus, and (iv) toxin-harboring pBAD33 with antitoxin-harboring pASK-IBA43plus. Both ara and AtC were used for induction. To test the toxicity of VapD proteins only, cells transformed with pBAD33-derived plasmids expressing VapD proteins were used.

### Tandem affinity purification

Several *E. coli* BL21(DE3) colonies transformed with pRSF_*hisVapD*_s*trepVapY-Mur*, pRSF_*hisVapD_strepVapW-Cje*, or pRSF_*hisVapD_strepVapW-Seq* plasmids were used to inoculate 10 mL of 2× YT supplemented with 50 µg/mL kanamycin. Cells were grown at 37°C until OD_600_ ~ 0.6. Protein expression was induced with 1 mM isopropyl-β-thiogalactopyranoside (IPTG) for 2 h at 37°C. Cells were resuspended in 1.5 mL of ice-cold buffer (50 mM Tris-HCl, pH 8.0; 250 mM NaCl, 3 mM imidazole, and 2 mM β-mercaptoethanol) supplemented with 0.2 mM phenylmethylsulfonyl fluoride. Cell lysis was performed by sonication. A total of 1,400 µL of cleared supernatant was mixed with 60 µL of Talon CellThru Co^2+^ chelating resin (Takara-Clontech) pre-equilibrated with the same buffer. The mixture was gently agitated for 2 h at 4°C. The resin was decanted and washed three times with the buffer, and bound proteins were eluted with the buffer supplemented with 0.5 M imidazole. The eluted material was combined with 60 µL of Strep-Tactin Super-flow Plus resin (Qiagen) pre-equilibrated with the same buffer. The mixture was gently agitated for 1 h at 4°C. The resin was decanted, washed two times with the buffer, and eluted with the buffer supplemented with 5.0 mM desthiobiotin (Sigma-Aldrich). Protein fractions were analyzed using SDS-PAGE.

### Protein expression and purification

For crystallization of the VapD-VapW complex, two proteins were co-expressed in *E. coli* BL21(DE3) Gold cells (Agilent) harboring the pRSF_*hisVapD*_*strepVapW-Cje* plasmid. Cells were grown at 37°C in 2 L of LB media supplemented with 30 mg/mL of kanamycin until OD_600_ 0.6–0.8. Protein expression was induced by the addition of 0.5 mM of IPTG, followed by growth at 18°C with 180 rpm shaking overnight. Cells were harvested by centrifugation, resuspended in lysis buffer (20 mM Tris-HCl, pH 8.0; 150 mM NaCl; 5 mM imidazole), and lysed by sonication. Cleared cell lysate was applied on 5 mL HisTrap HP column (Cytiva), and the VapD-VapW complex was eluted with the elution buffer 1 (500 mM imidazole, pH 7.8; 20 mM Tris-HCl, pH 8.0; 150 mM NaCl). The protein complex was further applied on Strep-Tactin Superflow Plus Cartridge (Qiagen), eluted with the elution buffer 2 (2.5 mM desthiobiotin; 20 mM Tris-HCl, pH 8.0; 150 mM NaCl), concentrated up to a final volume of 5 mL, and loaded on a HiLoad Superdex 75 16/600 column (Cytiva). The fractions from the main peak were mixed and concentrated up to 15 mg/mL for crystallization.

### Protein crystallization and structure determination

The VapD-VapW complex (0.2 µL) was mixed with an equivalent volume of the reservoir solution containing 20% (wt/vol) polyethylene glycol 1000; 100 mM sodium phosphate dibasic/citric acid, pH 4.2; and 200 mM lithium sulfate. Crystallization was performed by the sitting drop vapor diffusion technique at 20°C.

The X-ray diffraction data were obtained at the BL32XU of SPring8. The crystallization solution supplemented with 25% glycerol was used as a cryoprotectant. The data were processed with XDS software (48). The VapD-VapW complex belongs to space group C 2 2 21 with unit-cell dimensions *a* = 89.578, *b* = 94.807, and *c* = 137.684 and α = β = γ = 90°. The structure was solved by molecular replacement with PHENIX (49) using VapD-VapW complex structure prediction as a search model. The prediction was produced using AlphaFold v2.3.0 (50) in multimeric mode, employing default parameters and generating one prediction per model. The genetic databases utilized for the construction of the multiple sequence alignment and template search were synchronized on 4 March 2019. The highest-ranked model was used for crystal structure determination. The reliability of the model was assessed using pLDDT scores and PAE values. The final structure refinement was performed using PHENIX (49) and Coot (51) at 1.95 Å to *R*_work_ and *R*_free_ values of 0.221 and 0.249, respectively. Structures were visualized using UCSF ChimeraX 1.7 (52). Superimpositions of structures were performed with a matchmaker function with default options.

### SOS response time-course measurement

*E. coli* MG1655 Δ10 cells containing genomic deletions of 10 type II toxin-antitoxin systems (53) were transformed with pBAD33 with one of the toxin genes and reporter plasmid pSulA_RFP (54). Besides the TA deletions, the Δ10 strain bears several chromosomal rearrangements and single nucleotide polymorphisms (SNPs) (53). Freshly grown colonies were inoculated in 2× YT media containing glucose (1%), chloramphenicol (34 µg/mL), and ampicillin (100 µg/mL). After growth at 37°C until OD_600_ ~ 0.3–0.4 cells were collected and washed twice with 2× YT and resuspended in fresh 2× YT with 0.02% ara and no antibiotics to the final OD_600_ = 0.15. Two hundred microliter of each culture was transferred to a 96-well plate. The plate was placed into the EnSpire Multimode Plate Reader (Perkin Elmer) at 37°C. Cell culture OD_600_ and RFP fluorescence (553/574 nm) were collected every 15 minutes. Autofluorescence and optical density of sterile 2YT media were subtracted from the final values.

## RESULTS

### Two new protein families are associated with VapD homologs

**Fig 1:**
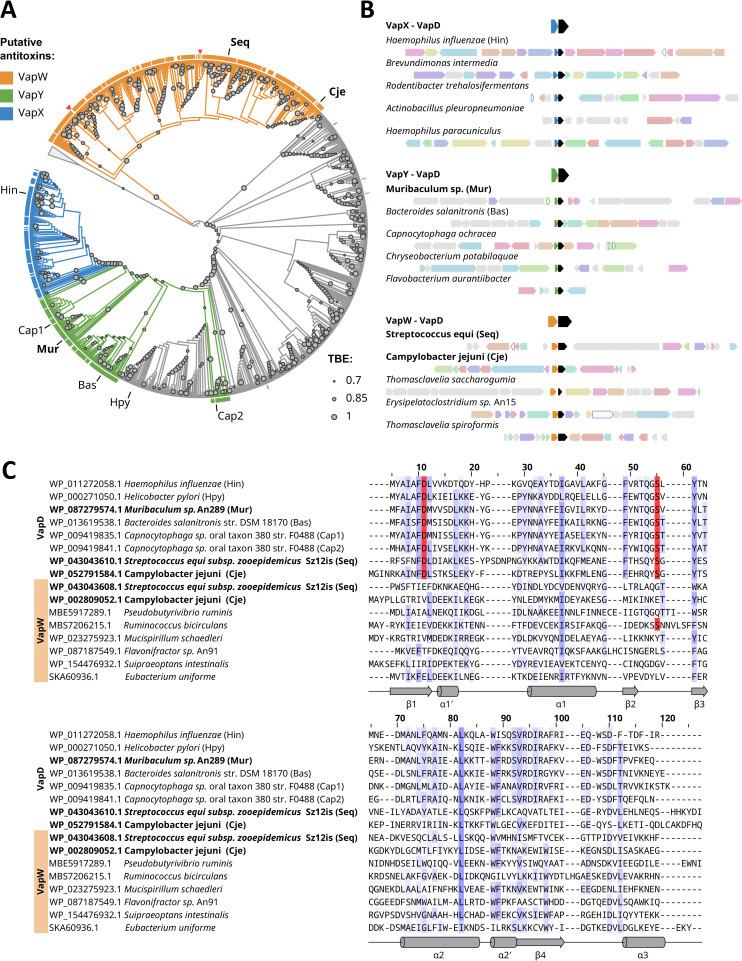
Phylogenetic analysis of VapD-like proteins. (**A**) A maximum-likelihood phylogenetic tree of VapD-like proteins. Transfer bootstrap expectation values are shown with gray circles on branches. Arcs on the outside indicate association with putative antitoxins. The arcs and VapD clades are colored according to a type of putative antitoxin (blue: VapX, green: VapY, and ocher: VapW). Red triangles outside the VapW arc indicate fused VapW-VapD proteins. Proteins are experimentally shown to be toxic and VapD^Hpy^ are labeled with abbreviated species names. Abbreviations of species harboring TA pairs characterized in this study are shown in bold. (**B**) Examples of genomic loci containing genes of VapD homologs. Genes are colored according to FlaGs clusterization (39). Genomic loci are labeled with the taxonomic name of the corresponding organism. Loci encoding proteins experimentally characterized in this study are highlighted with labels in bold font. (**C**) Multiple sequence alignment of VapD-like and VapW proteins. Sequences are labeled with NCBI protein accession numbers and taxonomic names. Labels of proteins experimentally characterized in this study are highlighted with bold font. Unaligned N- and C-terminal regions were omitted for clarity. Putative catalytic residues of VapD are highlighted with a red background. Shading intensity indicates the conservation of the position accounting for the BLOSUM62 score. Secondary structure elements are schematically shown based on the VapD^Cje^ crystal structure, which is described below.

To expand the family of VapD-like toxins and understand their evolution, we conducted an extensive search for VapD homologs in the NCBI nr database (33). The search was performed with PSI-BLAST using as a query a multiple sequence alignment of proteins from the UniProt database (29) annotated as VapD according to the PIRSF system (30). The resulting set of 2,727 proteins was clustered at 90% identity to remove redundancy, and 1,132 representative sequences were used to construct a phylogenetic tree (Fig. 1A).

To explore proteins associated with VapD homologs, we examined eight open reading frames (ORFs) upstream and downstream of each VapD-like gene in our set. Proteins encoded by *vapD*-adjacent genes were functionally annotated using the Pfam and TIGRFAMs databases (40, 41). Since many ORFs adjacent to genes encoding VapD homologs had no matching records in protein family databases, we performed sensitive clustering of proteins encoded in the vicinity of *vapD*-like genes to identify novel protein families using FlaGs (39).

VapD proteins encoded adjacent to genes of VapX antitoxin homologs (and thus representing TA systems of the known VapXD family) formed a compact clade on the tree (shown in blue in Fig. 1A). VapX-associated VapD proteins appeared to be a subclade of a larger clade, in which many *vapD* genes were adjacent to ORFs encoding proteins from a previously undescribed family (shown in green in Fig. 1A). This novel protein family was designated as VapY. VapD homologs from an even larger separate clade were associated with proteins from yet another family that we denoted VapW (shown in ocher in Fig. 1A). For the remaining VapD homologs (shown in gray in Fig. 1A, constituting ca. 50% of our data set), no association with specific proteins was observed.

Interestingly, multiple sequence alignment revealed that proteins from the VapW family are distant homologs of VapD proteins (Fig. 1C). However, at least one (usually both) of the catalytic aspartate or serine residues (17, 23) are mutated in VapW proteins, which should make them unable to catalyze nucleolytic reactions (Fig. 1C ; S1). The *vapD* and *vapW* genes appear to be prone to duplications: A-A-T-T (where A stands for *vapW*, a putative antitoxin gene, and T stands for *vapD*, the putative toxin gene), A-T-A-T-A-T, A-T-T, and other combinations were detected in our data set (Fig. S2).

### VapY and VapW function as antitoxins of cognate VapD toxins

Frequent co-localization of VapD homolog genes with genes coding for proteins of the two newly discovered families suggests that they may comprise toxin-antitoxin systems. We chose one VapY-VapD pair (from *Muribaculum* sp. An289 [Mur]) and two VapW-VapD pairs (from *Campylobacter jejuni* [Cje] and *Streptococcus equi subsp. zooepidemicus Sz12is* [Seq]) to validate this prediction. For each pair, genes coding for putative toxins and antitoxins were cloned into compatible plasmids pBAD33 and pASK-IBA43, respectively, allowing independent regulation of their expression. *E. coli* BW25113 cells co-transformed with both plasmids were grown in the presence of ara (induces toxin gene expression), AtC (induces antitoxin gene expression), or both, and the number of viable cells was determined by counting colony-forming units in aliquots of induced and uninduced cultures.

**Fig 2:**
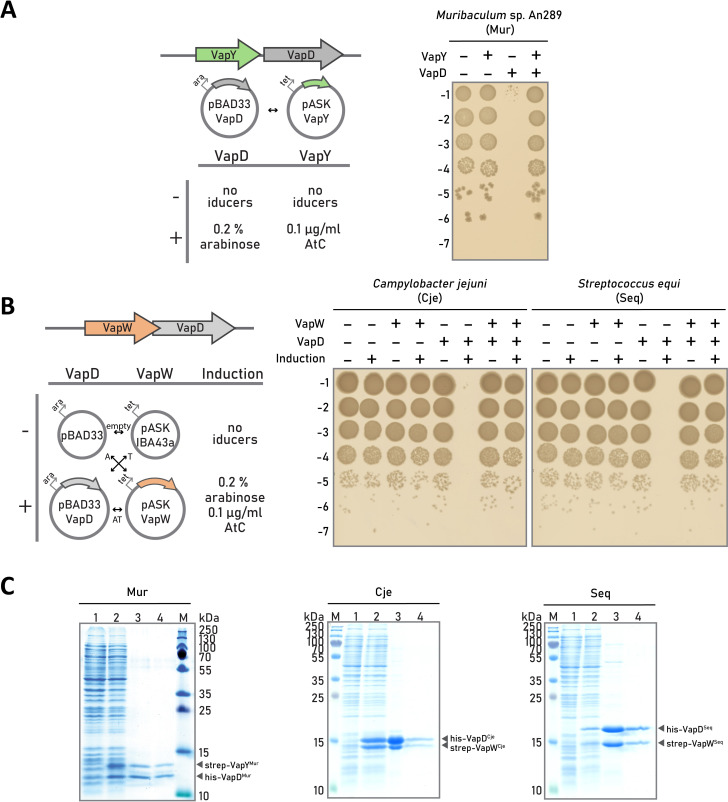
VapW-VapD and VapY-VapD are novel type II TA systems. (**A**) Validation of the VapD-VapY TA pair from *Muribaculum* sp. An289 (Mur). The putative antitoxin (*vapY*) and toxin (*vapD*) genes were cloned downstream of tetracycline and ara-inducible promoters, respectively. *E. coli* BW25113 were transformed with the resulting plasmids pBAD33_VapD and pASK_VapY, and toxin, antitoxin, or both proteins synthesis was induced by the addition of, respectively, 0.2% ara, 0.1 mg/µL of AtC, or both. Cultures were grown for 60 min in the presence or the absence of inducers, and aliquots of serial dilutions of the cultures were deposited on the surface of YT agar plates. Results of overnight growth at 37°C are shown. (**B**) Validation of VapW-VapD TA pairs from Cje and Seq. Since no effect was observed in a set-up used in panel **A** (Fig. S4), a different set-up was used to exclude the possibility of antitoxin production due to tetracycline promoter leakage. Putative antitoxin (*vapW*) and toxin (*vapD*) genes were cloned downstream of tetracycline and ara-inducible promoters, respectively. The resulting plasmids pBAD33_VapD (Seq or Cje) and pASK_VapW (Seq or Cje) or corresponding empty vectors pBAD33 or pASK_IBA43 were transformed in *E. coli* BW25113 in various combination. The viability of cells grown in the presence or in the absence of both inducers was next tested as in panel А but after 120 min of incubation. (**C**) Tandem affinity purification of 6×His-tagged VapD toxins and cognate strep-tagged VapY or VapW antitoxins from extracts of co-overexpressing cells. The protein content of samples corresponding to different steps of purification—before induction (lane 1), after induction (lane 2), material eluted from Talon Co^2+^-resin (lane 3), and material eluted from Strep-Tactin resin (lane 4)—was analyzed by SDS-PAGE and visualized by Coomassie staining. M, protein molecular weight markers.

Expression of *vapD*^Mur^ alone severely decreased cell viability, while expression of *vapY*^Mur^ had no such effect (Fig. 2A). Cells co-expressing *vapD*^Mur^ and *vapY*^Mur^ were as viable as cells in control uninduced cultures. Thus, the VapD^Mur^ and VapY^Mur^ proteins behave as a cognate toxin and antitoxin, respectively. Given that VapYD is a likely evolutionary predecessor of VapXD (Fig. 1A), to which the prototypical VapD toxin, VapD^Hin^, belongs, we tested three additional VapD proteins from the clade associated with VapY and found that all three were toxic (Fig. S3). We conclude that VapD homologs shown in green in Fig. 1A and their associated VapY proteins form a new VapYD family of toxin-antitoxin systems.

When the same validation strategy was applied to the VapW-VapD pairs, no effect on colony formation at conditions of *vapD* gene induction was observed (Fig. S4). We hypothesized that even in the absence of the AtC inducer, enough VapW antitoxin was produced due to tetracycline-inducible promoter leakage to neutralize the cognate toxins. Therefore, we employed a different validation scheme using empty pBAD33 or pBAD33_VapD and empty pASK-IBA43 or pBAD33_VapW plasmids (Fig. 2B). Almost no colony-forming units were observed in cultures where expression of the *vapD*^Cje^ or *vapD*^Seq^ genes alone was induced (Fig. 2B). Co-expression of cognate *vapW* genes alleviated toxicity. We, therefore, conclude that VapD homologs shown in ocher in Fig. 1A and their associated VapW proteins also form a novel VapWD family of toxin-antitoxin systems.

In type II TA systems, proteinaceous antitoxin neutralizes cognate antitoxin via direct binding. To check if VapY and VapW form complexes with their cognate VapD toxins, we performed tandem affinity purification from extracts of cells co-expressing hexahistidine-tagged VapDs and strep-tagged cognate antitoxins. As is shown in Fig. 2C, all three pairs of proteins co-eluted after tandem purification using Co^2+^-IDA and StrepTactin resins. We, therefore, conclude that VapYD and VapWD are type II toxin-antitoxin systems.

### VapD-like toxins induce the SOS response

**Fig 3:**
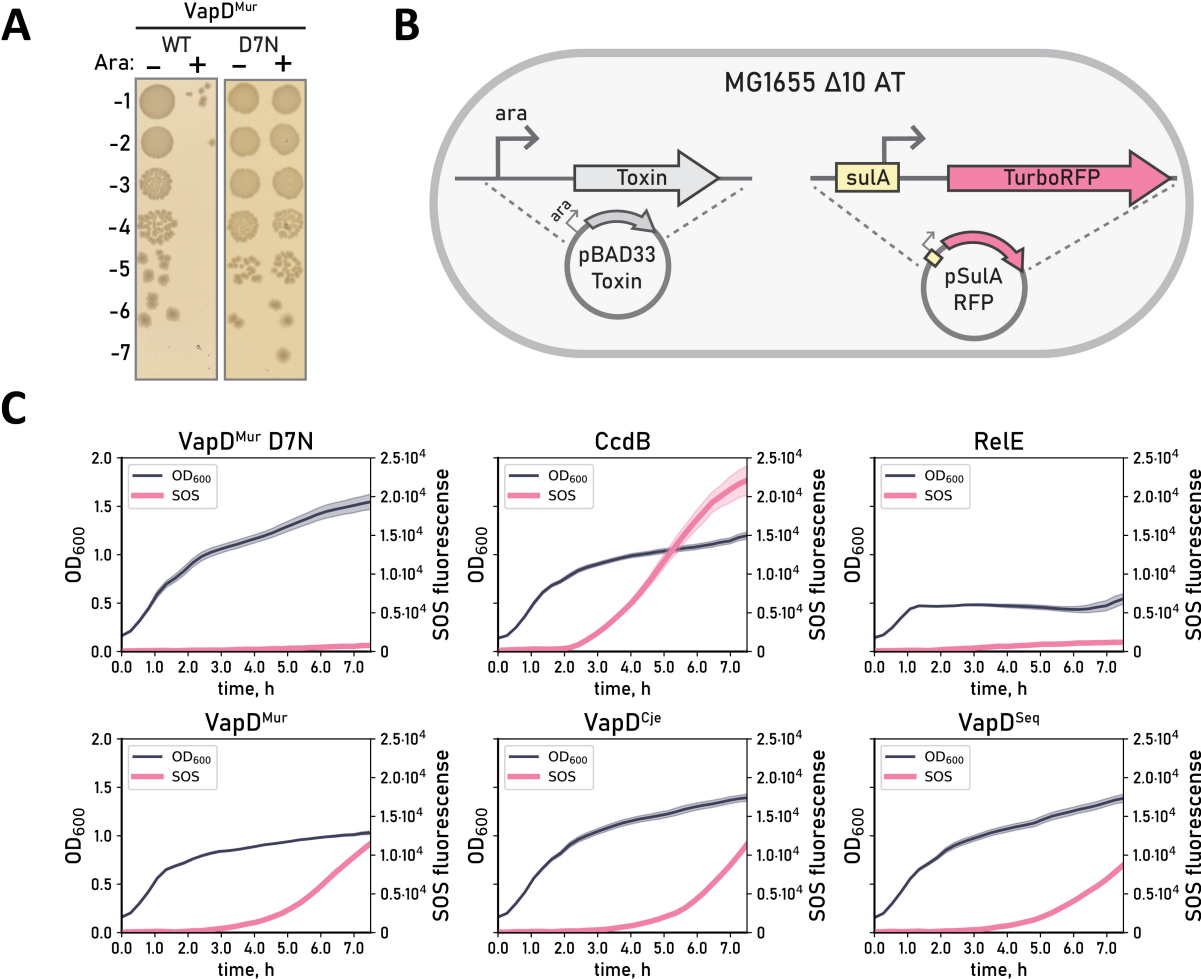
Expression of VapD toxins leads to the SOS response. (**A**) *E. coli* BW25113 was transformed with plasmids bearing wild-type or D7N inactive variant of the *vapD*^Mur^ gene under the control of ara-inducible promoter. Cells were grown for 60 min in the presence or the absence of ara, and aliquots of serial dilutions of the cultures were deposited on the surface of YT agar plates. Results of overnight growth at 37°C are shown. (**B**) Scheme of SOS response measurement assay. *E. coli* MG1655 Δ10 strain with 10 deleted TA systems was transformed with pBAD33 plasmid containing toxin gene (Tox) under the ara-regulated promoter and reporter plasmid pSulA-RFP containing fluorescent protein TurboRFP gene under the SOS-inducible sulA promoter (55). (С) Time-course measurement of optical density (OD_600_) and TurboRFP fluorescence of ara-induced cells from the experiment described in scheme B. An inactivated D7N VapD^Mur^ mutant was used as a negative control. The gyrase inhibitor CcdB was used as a positive control for SOS response. RNase RelE was used as a negative control.

The stand-alone VapD^Hpy^ and the VapD toxin from the VapXD^Hin^ TA system demonstrate non-specific RNase activity *in vitro* (16, 17). The Cas2 proteins are homologous to VapD (24) and were also reported to possess either RNase or DNase activity *in vitro* (25–28). We tested whether Asp7 of VapD^Mur^, a conserved residue corresponding to catalytic amino acids of VapD^Hpy^ and VapD^Hin^ (17, 23), is important for toxicity. As can be seen from Fig. 3A, expression of the D7N VapD^Mur^ single substitution mutant did not affect cell viability, implying that toxicity of VapD^Mur^ might be caused by its nuclease function.

We tested the *in vivo* effects of VapD overproduction using the pSulA-RFP reporter plasmid with a turboRFP gene placed under the control of an SOS-inducible promoter (54). When cells harboring pSulA-RFP undergo SOS response, the turboRFP fluorescent signal is increased (Fig. 3B). As expected, in cultures of cells carrying pSulA-RFP and a compatible plasmid expressing CcdB, a toxin that inhibits the DNA gyrase (56), a strong increase in fluorescence was observed after 2 h post-induction (Fig. 3C). This effect was specific to the SOS response since no turboRFP fluorescence increase was observed with RelE (Fig. 3C) and several other translation-inhibiting toxins when overproduced (Fig. S5). The expression of VapD^Mur^ led to increased fluorescence 3 h post-induction, while the expression of catalytically inactive and non-toxic D7N VapD^Mur^ mutant did not (Fig. 3C). We conclude that VapD^Mur^ causes SOS response, likely through its nucleolytic function. Expression of VapD^Cje^ and VapD^Seq^ had a similar effect. Replication inhibition by VapD toxins was further confirmed by cell filamentation upon overproduction of VapD^Mur^ (Fig. S6). Further studies are required to determine whether replication inhibition is directly caused by the DNase activity of VapD toxins.

### Crystal structure of the VapD-VapW complex

To elucidate the interaction between VapD and the newly discovered antitoxins, we attempted to crystallize several toxin-antitoxin complexes prepared from co-overexpressing cells. Diffracting crystals were obtained only for the VapD^Cje^*-*VapW^Cje^ pair. The structure was refined to an *R* factor of 0.221 and *R*_free_ of 0.249 at 1.95 Å resolution. A detailed summary of data collection and refinement statistics is provided in Table S4. The asymmetric unit contains two VapD and two VapW molecules (Fig. S7A). The protein chains are nearly complete, except for sections of 20 and 25 residues at the C-terminus of the two VapD chains that are not resolved. Additionally, one of the toxin chains lacks four residues within the putative catalytic cleft, and one of the antitoxin chains lacks the C-terminal residue.

**Fig 4:**
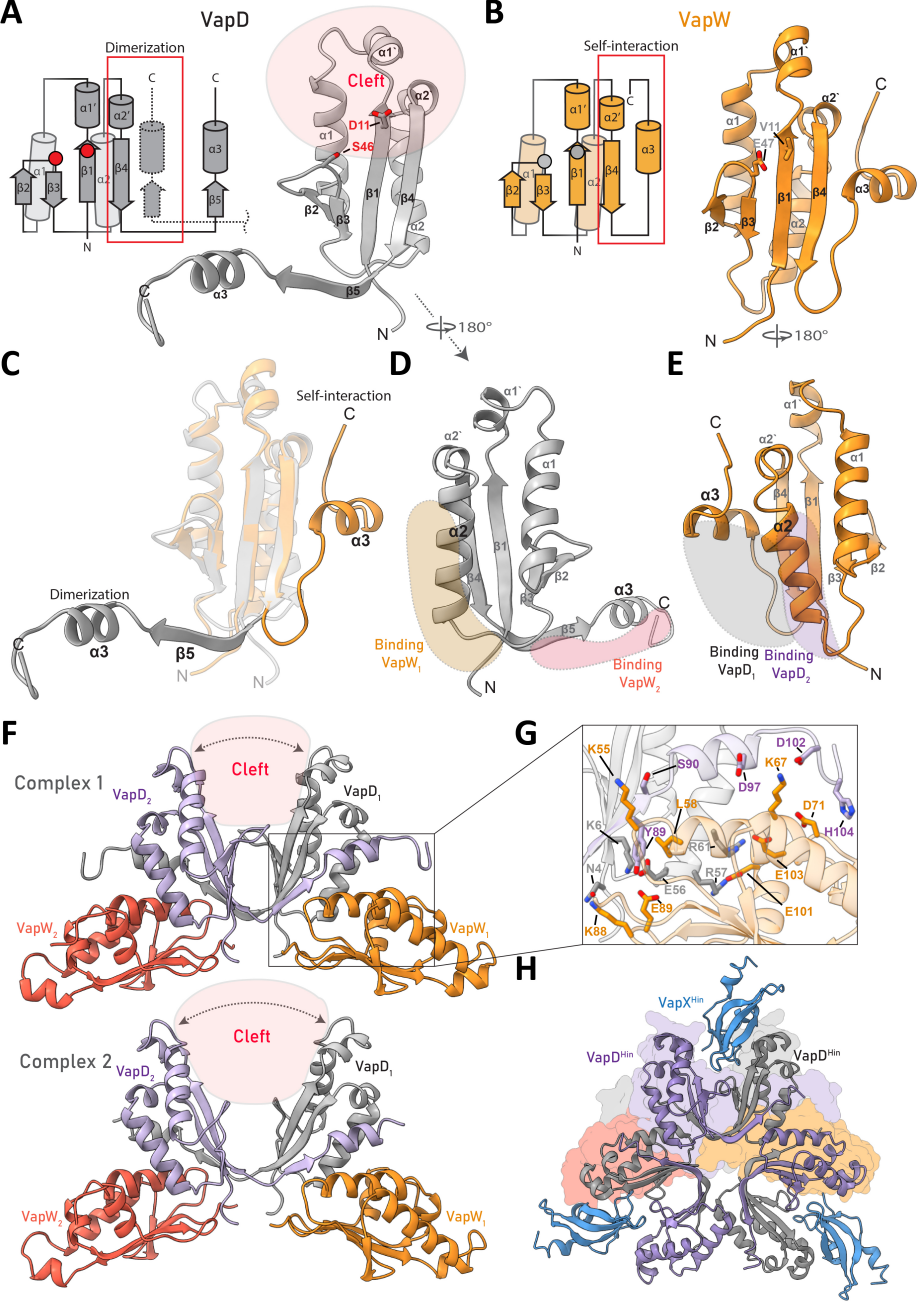
Structure of the VapD-VapW complex. (**A**) Structure of VapD^Cje^ from VapD-VapW complex 1 (right) and its topology diagram (left). The red rectangle represents the dimerization interface with the C-terminus of another VapD subunit, shown with dashed lines. Two catalytic residues, D11 and S46, are shown on the structure and indicated with two red circles on the diagram. The red area on the structure represents the location of the catalytic cleft that is formed between two VapD^Cje^ subunits. (**B**) Structure of VapW^Cje^ from the VapD-VapW complex 1 (right) and its topology diagram (left). The red rectangle highlights the interaction between the C-terminus and the core region of the protein. Residues V11 and E47, which correspond to catalytic residues D11 and S46 of VapD, are shown on the structure and designed with two gray circles on the diagram. (**C**) VapW^Cje^ (orange) and VapD^Cje^ (gray) structures superimposition. (**D**) Binding sites of VapD^Cje^ to two subunits of VapW^Cje^. (**E**) Binding sites of VapW^Cje^ to two subunits of VapD^Cje^. (**F**) Two kinds of VapD-VapW complexes in the crystal with different distances between VapD subunits. The catalytic cleft is shown in red. (**H**) Crystal structure of VapD-VapX from *H. influenzae* (PDB ID: 6ZN8). The figure in the background represents the surface of the VapD-VapW complex 1.

As expected, both VapD^Cje^ and VapW^Cje^ adopt a modified ferredoxin-like fold (β1α1′α1β2β3α2α2′β4) characteristic of VapD and Cas2 proteins (Fig. 4A and B) (23). In addition to the ferredoxin-like core, proteins of both families possess an extended C-terminal region involved in homodimerization. VapD^Cje^ and VapW^Cje^ are no exception, both having an extended C-terminus with an additional α3 helix (Fig. 4A and B). However, while the α3 helix of VapD^Cje^ interacts with the core of another VapD subunit, the α3 of VapW^Cje^ interacts with its own core. As a result, VapW does not form homo- and heterodimers the way VapD and Cas2 do.

While the two VapD molecules in the asymmetric unit do not form a homodimer (Fig. S7A), each of them forms homodimers related by a crystallographic twofold axis, resulting in two distinct VapD-VapW complexes with a 2:2 stoichiometry, designated as complexes 1 and 2 (Fig. 4F and 7A). Consistent with the 2:2 stoichiometry, a single peak corresponding to 60 kDa was detected during mass photometry measurements (Fig. S7B). In both complexes, the VapD^Cje^ molecules adopt an open conformation analogous to those of VapD^Hin^ bound to cognate VapX antitoxin (23) and of VapD^Hpy^ (17) (Fig. 4H; Fig. S7C). Good superimpositions of VapD^Cje^ monomer with the VapD^Hin^ and VapD^Hpy^ structures (RMSD of Cα atoms 0.9 Å and 1.2 Å, respectively; Fig. S7D) highlight the high conservation of the protein fold. In the case of complex 2, the distance between the two VapD subunits is larger, which likely makes the loop in the catalytic cleft between β2 and β3 more flexible and disordered in the crystal.

### Interaction of VapD and VapW

While the VapX antitoxin binds in the cleft between the two cognate VapD subunits and occludes the catalytic site (23) (Fig. 4H), VapW binds on the opposite side of the VapD enzyme. Previously, VapD^Hin^ was shown to possess a second oligomerization interface that allows it to form a higher-order structure with three dimers assembled in a hexameric ring (23). In the VapD-VapW complex, the binding of the VapD toxin to the VapW antitoxin mimics the interactions between the dimers in the VapD^Hin^ hexamer (Fig. 4F and H). Consequently, VapW should prevent the VapD hexamer formation; however, further studies are needed to determine whether VapDs from the new group can form higher-order structures and, if so, whether it is relevant to their activity.

Each VapW chain simultaneously interacts with both VapD subunits (Fig. 4D and E). The first VapD subunit interacts with VapW primarily via the α2 helix, whose N-terminus is embedded into the cavity formed by the α2 helix and the β4-α3 region of the antitoxin. Here, the positively charged patch on the surface of VapD binds the negatively charged area of VapW. The patch of VapD is formed by residues R57 and R61 that form salt bridges with the E101 and E103 of VapW, respectively, with E101 being sandwiched between the two arginines. The backbone nitrogen of E56, the first residue of the α2 helix of VapD, forms a hydrogen bond with the E89 of VapW. Additionally, the N-terminal residues N4 and K6 of VapD interact with K88 and E89 at the loop between β4 and β5 of VapW (Fig. 4G).

The second VapD subunit interacts with the antitoxin via its C-terminal end, where the α3 helix of VapD lies parallel to the α2 helix of VapW (Fig. 4D, E and G). Interactions in this area occur between the end of the α2 helix of VapW and the unstructured C-terminal end of VapD. The side chain of H104 in VapD forms a hydrogen bond with the backbone of D71 in VapW. The side chain of K67 in VapW interacts with the side chain of D102 and the backbone oxygen of D97 in VapD. However, these interactions are present only in complex 1, suggesting that the residues involved may be flexible, and this interaction is not essential for complex formation. Another patch of contacts is located on the end of the β4 strand of VapD. The backbones of S90 in VapD and K55 in VapW form a hydrogen bond. Y89 in VapD engages in hydrophobic interaction with L58 in VapW. Additional possible interactions involving the 20 most C-terminal residues of VapD cannot be identified as they are not resolved in the structure.

## DISCUSSION

In this work, we explored the diversity of VapD toxin homologs and associated genes. This approach led to the discovery of two novel antitoxin families (VapY and VapW), expanding the range of known VapD-associated antitoxins beyond the canonical VapX. Phylogenetic analysis revealed that VapX-associated toxins are a subclade of VapDs that pair with VapY antitoxins. The AlphaFold Protein Structure Database (57, 58) contains a high-confidence structure prediction of VapY (ID: AF-A0A1Y4C551-F1, pLTTD score 96.69). Surprisingly, VapY appears to have an SH3-like beta-barrel fold similar to that seen on the VapX structure previously determined in complex with VapD (23) (Fig. S8A through CFig. S8A through C). However, in addition to the low conservation of amino acid sequence (Fig. S8D), VapX and VapY antitoxins have their own characteristic structural features. While VapY has a classical five β-strands architecture, in VapX, the β5 strand is reduced to just two residues. Moreover, VapY, in contrast to VapX, has an α1-helix between β1 and β2 strands and lacks the α2-helix on the C-terminal end. These differences make independent acquisition of these antitoxins by ancestral closely related VapY the most plausible evolutionary scenario. For example, toxins of this subfamily at some point may have acquired a different antitoxin due to the loss or malfunction of the original antitoxin gene.

Surprisingly, the VapW-associated VapD proteins are phylogenetically distant from the VapX/VapY clade, while more closely related VapD-like proteins lack identifiable antitoxins. This indicates that the two subfamilies of TA systems based on VapD-like toxins, one associated with VapX/VapY and another associated with VapW, may have emerged independently. Sequence and structure similarity indicate that VapW antitoxins are inactive homologs of VapD itself. A feasible evolutionary scenario involves the duplication of a VapD gene followed by a subsequent functional transition. This hypothesis is supported by the conservation of structural features and the mode of VapD^Cje^-VapW interaction, which is similar to the interaction of two VapDs forming a higher-order ring structure, as previously shown for VapD^Hin^ (23). The functional transition from antitoxin to toxin was previously described for the SymE ribonuclease (59), an AbrB superfamily protein that has the same fold as the MazE antitoxin and whose homologs were shown to act as antitoxins of MenT toxins (60). YeeU is another example of an antitoxin that shares a fold with toxins ParE and RelE (61). However, toxin gene duplication with subsequent inactivation of one of the copies was not previously shown, even though such a mechanism seems to be an obvious way of TA pair formation for toxins that function as oligomers.

Several organisms possess multiple copies of the VapWD system (Fig. S2). The evolutionary mechanism(s) driving the expansion of VapWD copy numbers remain unclear. For such duplication to persist, it should confer a selective advantage that mitigates the associated fitness cost (62). When present, the observed multiple copies of VapWD are adjacent and likely have not evolved distinct regulation mechanisms or functions. In principle, the increased toxin dosage caused by duplications could provide a more rapid response to phage predation or other environmental stresses. Given that a number of bacteria harboring multiple copies of VapWD are associated with the gastrointestinal tract, such duplications might offer a survival benefit specific to this highly crowded and phage-rich environment. For example, the VapWD system could help to withstand the bile acid stress, similarly to the MqsRA system (63).

The VapW antitoxins could have appeared through similar duplication events. While selective pressure maintaining the duplication should counter the inactivation of one of the VapD copies (a challenge known as Ohno’s dilemma [64]), in the absence of selection, one copy of VapD would quickly accumulate deleterious mutations leading to VapW. Alternatively, the duplicated copies of VapWD may have reduced activity and/or expression levels, and to compensate for this and maintain the original function, all of them are needed, following a process of subfunctionalization (65). If this scenario occurred during VapW evolution, it might explain the accumulation of mutations leading to the eventual inactivation of one copy.

The mechanism of VapD toxicity remains enigmatic. The observation that VapD proteins from new TA pairs induce SOS response suggests DNA replication and/or genome maintenance as a primary target of these toxins. The stand-alone VapD^Hpy^ (*H. pylori*) and VapD^Hin^ (*H. influenzae*) from the VapXD TA system were previously reported to exhibit RNase activity *in vitro*. The VapD nucleases may possess both DNase and RNase activities simultaneously, as has been demonstrated for some Cas2 proteins (26–28). Conversely, it is possible that the SOS response induced by VapD is due to the inhibition of protein(s) involved in DNA maintenance, as was described for CcdB (inhibits DNA gyrase) (56) and SocB (inhibits clamp proteins) (66). CcdB is a prime example of a functional transition from translation inhibition to replication inhibition, as the structurally similar CcdB toxin MazF possesses RNase activity. However, protein-protein interaction is a somewhat unlikely mechanism of VapD protein toxicity, given that they are functional in orthologous organisms, and the presumed catalytic residues are required for toxicity/induction of the SOS response.

The crystal structure of the VapD-VapW complex indicates that VapW neither blocks the catalytic cleft of VapD^Cje^ nor disrupts homodimer formation. This distinguishes the VapD-VapW complex from most type II TA systems, where toxin neutralization is typically achieved via active site hindrance. Although unusual, several similar cases have been previously described. For example, toxin inactivation can be achieved through allosteric regulation, as observed in the HipB-HipA system, where the HipB antitoxin secures an inactive conformation of the toxin (67). The interaction interface of the ribosome-dependent RNase toxin HigB with cognate antitoxin HigA is also located remotely from the catalytic site. It has been proposed that the antitoxin sterically prevents toxins’ interaction with the ribosomally-bound mRNA (68). A similar mechanism of inhibition is observed in the anti-CRISPR protein AcrVA5, which interacts with the VapD homolog Cas2 and prevents its interaction with DNA protospacers (69) (Fig. S9). AcrVA5 interacts with the α2 helix of Cas2, near the region that corresponds to the VapW interaction interface in VapD. Although the formation of the higher-order structure was demonstrated only for VapD^Hin^ and its functional role remains unclear, impairment of the higher-order structure by VapW binding may also contribute to the regulation of VapD^Cje^ activity. To fully understand the mechanism of VapD neutralization by VapW and the mechanism(s) of VapD action, further research on the cellular targets will be needed.

## Data Availability

The data set of all identified VapD homologs and the results of the adjacent genes analysis are provided in Table S1. The crystal structure of the *C. jejuni* VapD-VapW toxin-antitoxin complex is deposited to PDB under the accession number 9KAN.

## SUPPLEMENTAL MATERIAL

Supplemental material mbio.00003-25-s0001.pdf Supplemental methods, Figures S1 to S9, and Tables S2 to S4.

Table S1 mbio.00003-25-s0002.xlsx All identified VapD homologs and the results of the analysis of the adjacent genes.

ASM does not own the copyrights to Supplemental Material that may be linked to, or accessed through, an article. The authors have granted ASM a non-exclusive, world-wide license to publish the Supplemental Material files. Please contact the corresponding author directly for reuse.
