# Supplementary material for: Novel type II toxin-antitoxin systems with VapD-like proteins
Source: mBio. 2025 Mar 7;16(4):e00003-25. doi: 10.1128/mbio.00003-25 (PMC11980593; doi:10.1128/mbio.00003-25)
Supplement: Supplemental material — Supplemental methods, Figures S1 to S9, and Tables S2 to S4. [file mbio.00003-25-s0001.pdf]

SUPPLEMENTARY METHODS

Plasmid construction

Except for *vapD*^{Mur} and *vapY*^{Mur}, Evrogen (Russia) delivered *vapW* and *vapD* genes pre-cloned into specified vectors using the protocol described below. To construct plasmids for toxicity tests and other assays *vapD, vapW,* and *vapY* were PCR amplified using appropriate primers. The resulting fragments harboring an rbs-containing ggagaatctcta sequence were inserted via XbaI and HindIII restriction sites into pBAD33 (for VapD) or pASK-IBA43plus (for VapY and VapW) plasmid vectors. The same plasmids were also used for the SOS-response measurements. Vectors used as positive (pBAD33_*ccdB*) and negative (pBAD33_*relE*) controls were constructed similarly.

To fuse VapY and VapW antitoxins with N-terminal Strep-tag, fragments containing PCR amplified genes were treated with BamHI and XhoI restriction endonucleases and inserted in the pET22(b) shuttle vector (Novagen-Millipore) treated with the same enzymes. These constructs were further digested with NdeI and XhoI restriction enzymes and resulting *strepVapY* and *strepVapX* fragments were introduced into the pRSF-Duet1 plasmid. Toxin genes were PCR-amplified using the corresponding pBAD33 constructs as templates and pBAD_rev primer (gatttaatctgtatcagg) as reverse PCR primer, treated with BamHI and HindIII and inserted into the pRSF_*strepVapY* or pRSF_*strepVapW* plasmid treated with the same restriction enzymes. The resulting plasmids were designated as pRSF_*hisVapD_strepVapY-Mur* (for *Muribaculum* sp. An289 TA pair), pRSF_*hisVapD_strepVapW-Cje* (for *Campylobacter jejuni* TA pair*),* pRSF_*hisVapD_strepVapW-Seq* (*Streptococcus equi subsp. zooepidemicus Sz12is)*.

Microscopy

Cells harboring the pBAD33-derived plasmid were grown and induced as described for the toxicity test. Aliquots of the cell cultures were taken before the addition of arabinose (0 hours) and after 1, 3, and 6 hours of incubation with arabinose. 1-μL aliquots were applied to agarose pads (1.2% agarose in PBS) and imaged using a Nikon Eclipse Ti microscope equipped with a Nikon Plan Apo VC 100X/1.40 oil objective and a Nikon DS-Qi2 digital monochrome camera. Images were processed using ImageJ software (1).

DNA extraction

Cells harboring pBAD33-derived plasmid were grown and induced as described for the toxicity test, except that the incubation time after the addition of arabinose (ara) was extended to 4 hours. Cells were then harvested by centrifugation, and DNA was extracted using the GeneJET Genomic DNA Purification Kit (ThermoFisher Scientific), following the manufacturer's protocol. DNA integrity was assessed by electrophoresis in 0.5 % agarose gel.

Mass photometry

VapD-VapW complex was diluted with a lysis buffer (20 mM Tris-HCl, pH 8.0; 150 mM NaCl; 5 mM imidazole) to an approximately 20 nM concentration. TwoMP mass photometer (Refeyn) was used according to the standard protocol (2). Mass photometry optics was focused onto 10 μL of lysis buffer droplet, and 10 μL of the diluted sample was added and mixed with the buffer in the same cell. The recording was performed immediately after mixing for 60 seconds using AcquireMP software. MP signals were calibrated using BSA and human IgC solutions (Maruwa) and the data were processed using DiscoverMP software.

SUPPLEMENTARY FIGURES

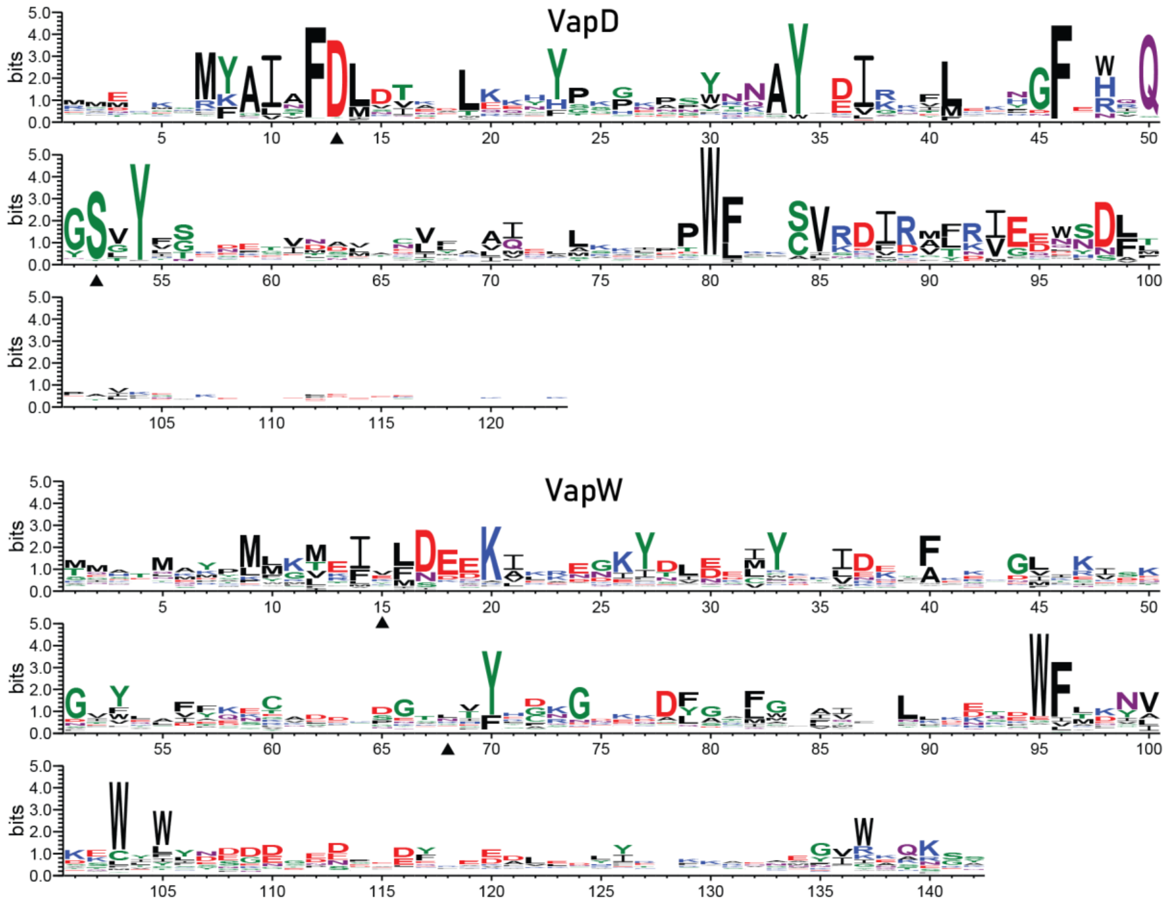

Figure S1. Conservation of VapD and VapW sequences. Logos of multiple alignments of VapDs and VapWs. Triangles under the horizontal axis indicate positions corresponding to the putative catalytic residues in VapD.

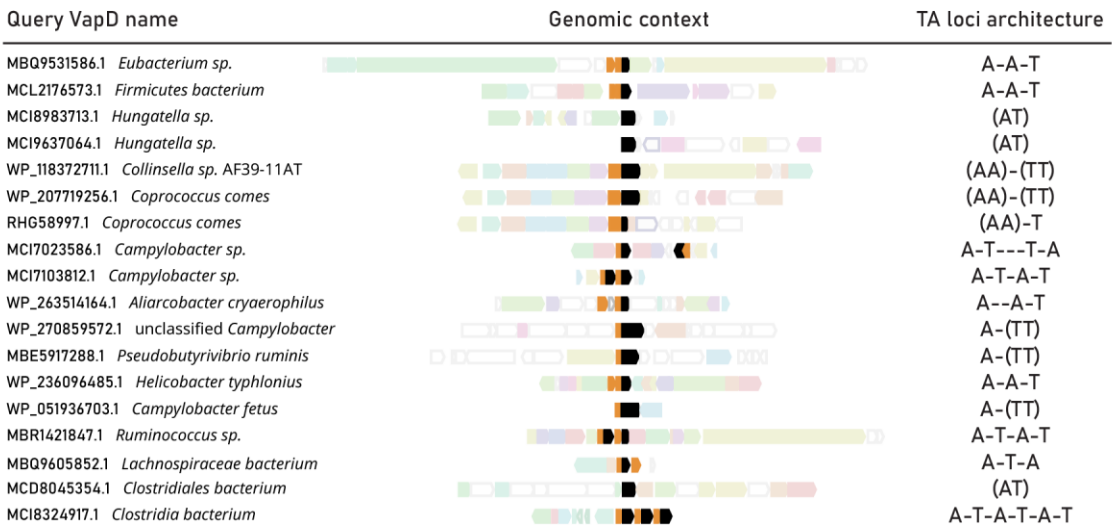

Figure S2. Examples of duplications and fusions of *vapD* and *vapW* genes. The first column contains the VapD protein accession number in the nr database and the species name of the corresponding bacteria. The second column represents the genomic context of the *vapD* gene. *vapD* is shown in black and *VapW* in orange. The third column is a schematic representation of the *vapD* (T) and *vapW* (A) genes organization. Fusion variants are enclosed in parentheses.

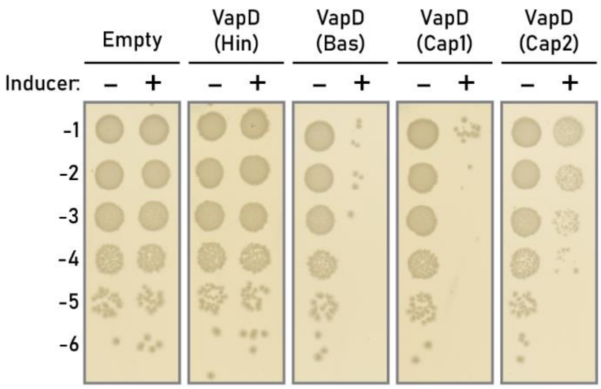

Figure S3. Toxicity of VapD toxins. Serial dilutions of *E. coli* BW25113 cells cultures harboring empty pBAD33 vector or pBAD33 with with indicated cloned *vapD* toxins genes were grown for 60 min in the presence or the absence of the inducer (0.2% arabinose). Tested toxins included *Haemophilus influenzae VapD* (VapD^{Hin}) associated with a VapX antitoxin (3) and VapY-associated VapD homologues from *Bacteroides salanitronis* str. DSM 18170 (VapD^{Bas}) and from *Capnocytophaga* sp. oral taxon 380 str. F0488 (VapD^{Cap1} and VapD^{Cap2}).

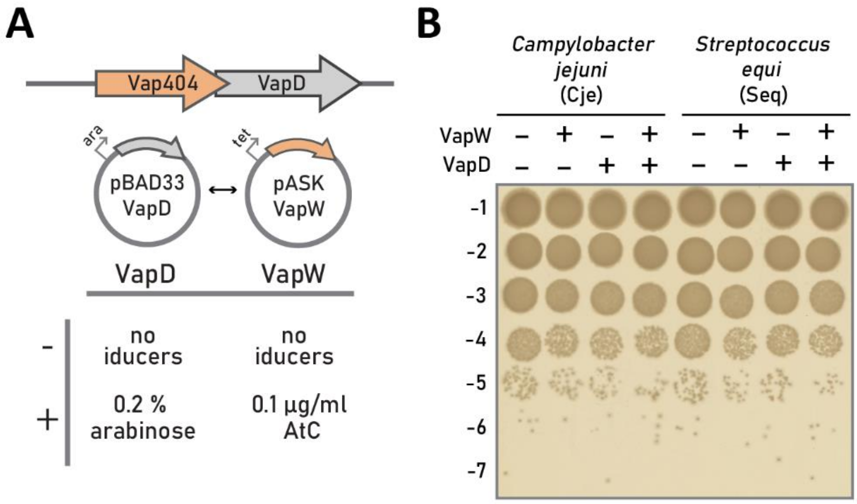

Figure S4. Leaky expression of *vapW* neutralizes VapD toxins. A) Scheme of toxin TA pair validation. The putative antitoxin (*vapW*) and toxin (*vapD*) genes were cloned downstream of tetracycline and arabinose-inducible promoters, respectively. *E. coli* BW25113 were transformed with the resulting plasmids pBAD33_VapD and pASK_VapY and toxin, antitoxin, or both proteins synthesis was induced by the addition of, respectively, 0.2 % arabinose, 0.1 mg/µl of anhydrotetracycline (AtC), or both. Cultures were grown for 60 min in the presence or the absence of inducers and aliquots of serial dilutions of the cultures were deposited on the surface of YT agar plates. Results of overnight growth at 37 °C are shown. **B)** Serial dilutions of the *E. coli* BW25113 cell as described in the scheme **A**. No effect on colony formation was observed. We hypothesized that antitoxin could be produced due to tetracycline promoter leakage.

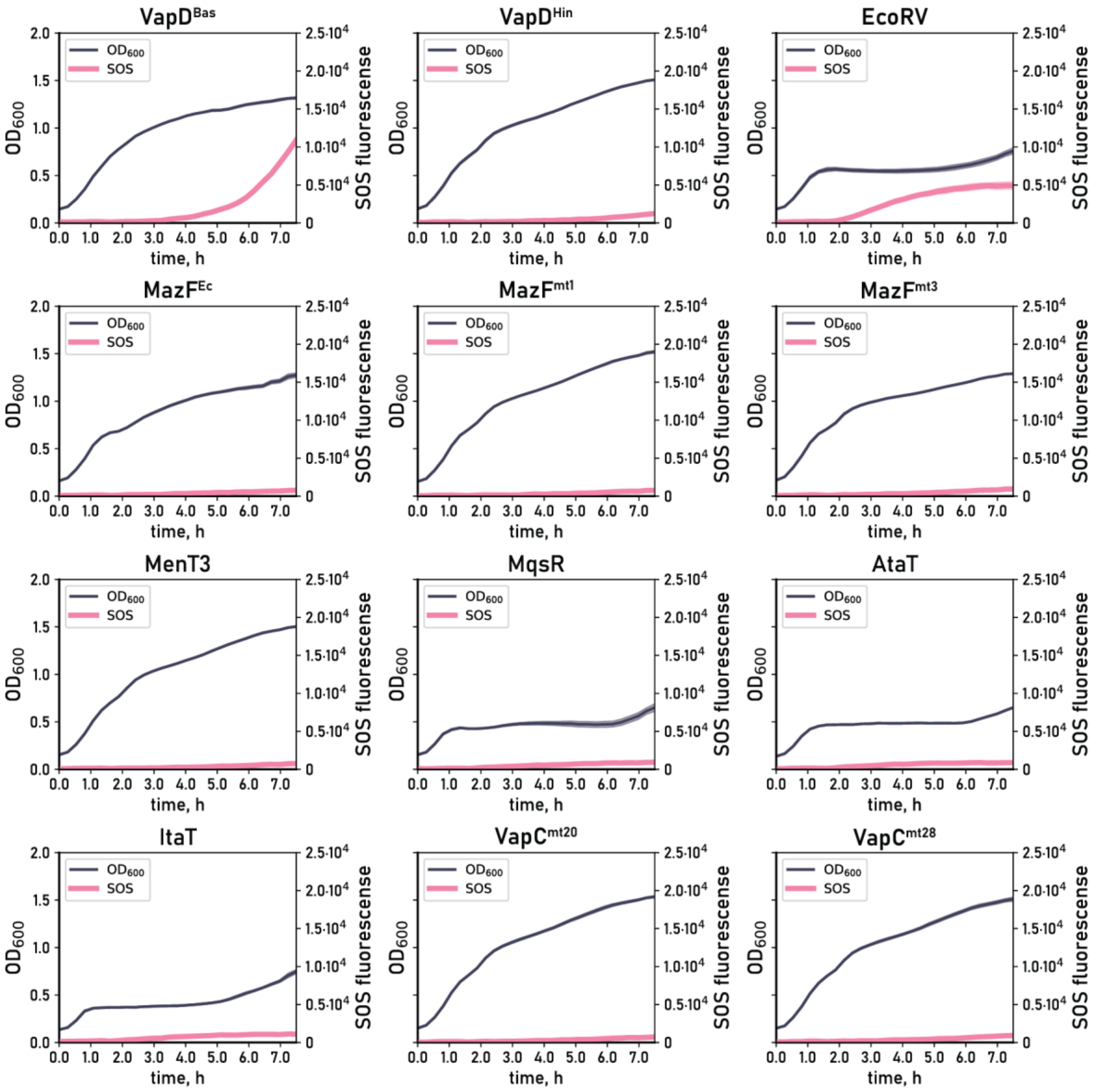

Figure S5. SOS response in cells expressing various toxins. Time-course measurement of optical density (OD$_{600}$) and TurboRFP fluorescence in cultures of arabinose-induced *E coli* MG1655 strain with deleted ten endogenous TA systems transformed with a pBAD33-based plasmid containing toxin gene under control of arabinose-regulated promoter and a reporter plasmid pSulA-RFP with the fluorescent protein TurboRFP gene under control of the SOS-inducible *sulA* promoter. Tested toxin genes encode VapD^{Hin} from *Haemophilus influenzae* (4), VapD^{Bas} from *Bacteroides salanitronis* str. DSM 18170 (this work); restriction endonuclease EcoRV (5); RNAses MazF^{mt1}, MazF^{mt3} (6), VapC^{mt20}, VapC^{mt28} (7); tRNA terminal nucleotidyltransferase MenT3 (8) from *Mycobacterium tuberculosis* H37Rv; RNases MazF^{Ec} (9) and MqsR (10) from *E. coli* K-12; and GNAT toxins AtaT from *E.coli* O157:H7 (11) and ItaT from *E.coli* HS (12).

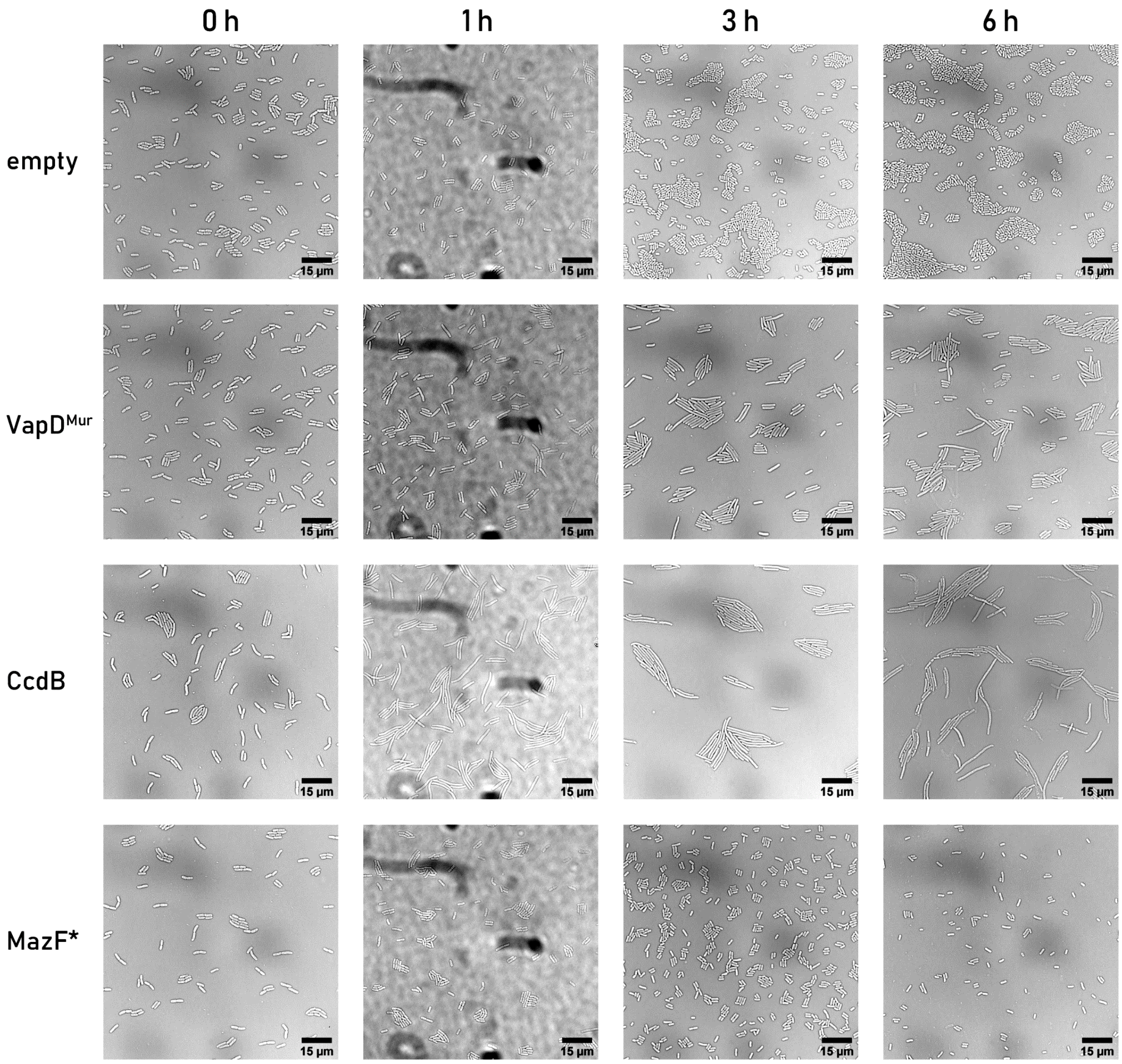

Figure S6. Cell filamentation upon expression of different toxins. Micrographs of *E. coli* BW25113 transformed with pBAD33-derived plasmids containing different toxin genes. Cells were induced with arabinose for varying times.

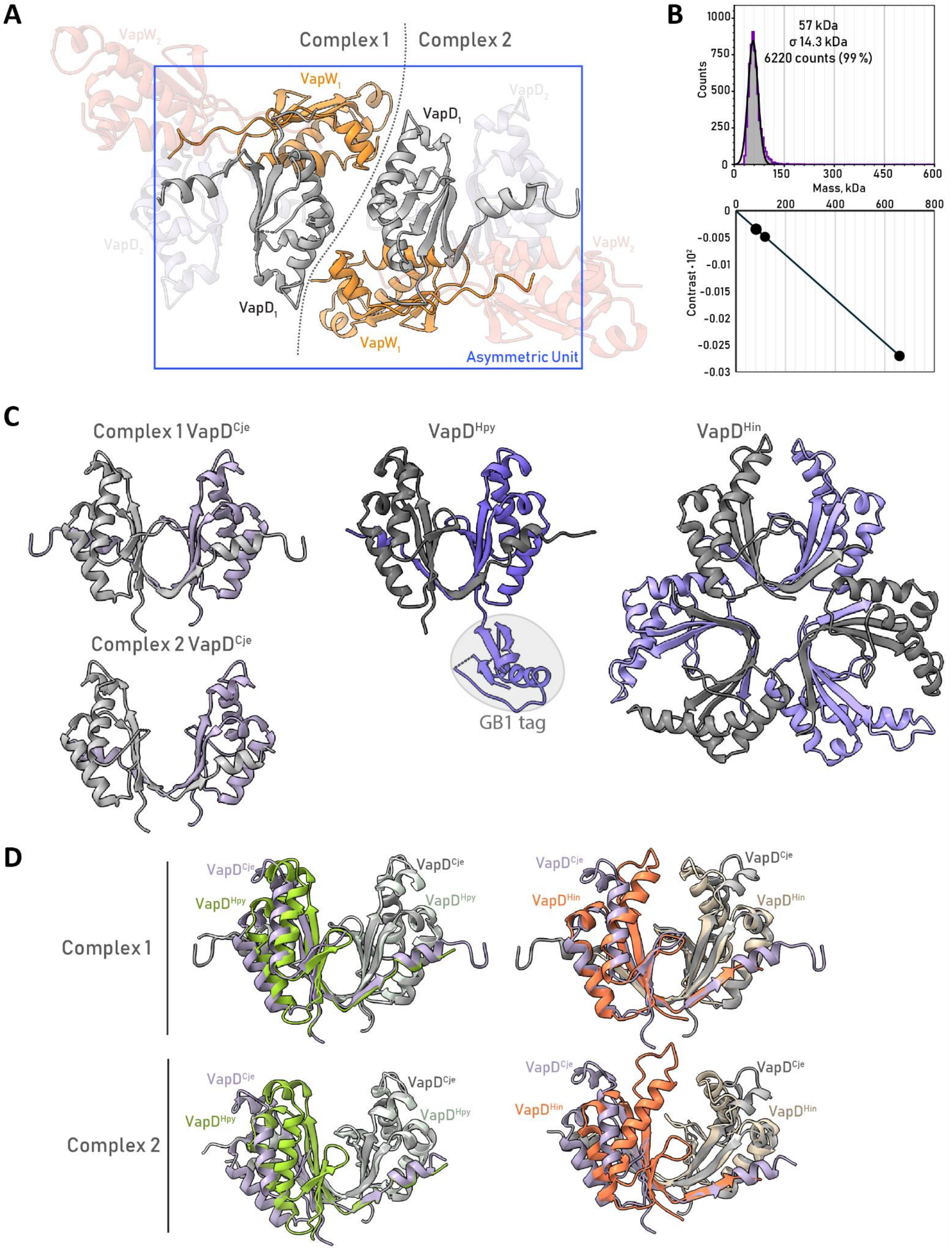

Figure S7. Structure of the VapD-VapW complex. A) Asymmetric unit of VapD^{Cje}-VapW^{Cje} crystal structure. The dashed line separates subunits that belong to complex 1 or complex 2. **B)** Mass-photometry measurement of VapD^{Cje}-VapW^{Cje} complex. The upper panel shows the mass histograms of the purified VapD^{Cje}-VapW^{Cje} complex (gray). The Gaussian fitting is shown in a black line. The lower panel shows the contrast to mass calibration used for the complex molecular weight determination. BSA

(66 kDa and 132 kDa) and human IgC (132 kDa) solutions were used as calibrants. **C)** Different VapD complexes. From left to right: Complex 1 and 2 of VapD^{Cje}-VapW^{Cje} with hidden chains of VapW^{Cje}; VapD^{Hpy} HP0135 from *H. pylori* tagged with GB1 tag, highlighted with the gray area (PDB ID: 3UI3); VapD^{Hin} from *H. influenzae* (PDB ID: 6ZI0). **D)** Superimposition of the two VapD^{Cje} complexes with previously described structures of VapD^{Hpy} and VapD^{Hin}.

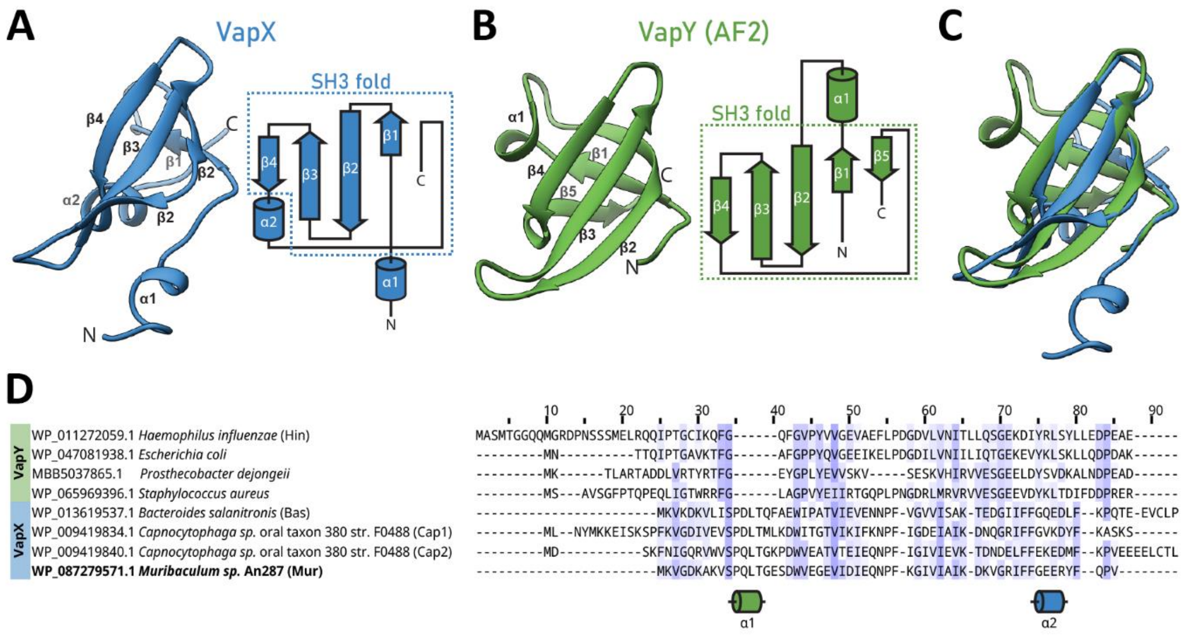

Figure S8. Similarity between VapY and VapX antitoxins structures. A) Structure of VapX^{Hin} antitoxin (*Haemophilus influenzae*) from the VapD-VapX complex PDB ID: 6ZN8 (left) and its topology diagram (right). **B**) Predicted structure (AlphaFold Protein Structure Database ID: AF-A0A1Y4C551-F1) of VapY^{Mur} (*Muribaculum* sp. An289) (left) and its topology diagram (right). **C**) VapX (blue) and VapY (green) structures superimposition. **D**) Multiple sequence alignment of several example VapX and VapY protein sequences based on structural superimposition. Secondary elements that are present only in one of the two protein families are shown below and colored according to family (blue - VapX, green - VapY).

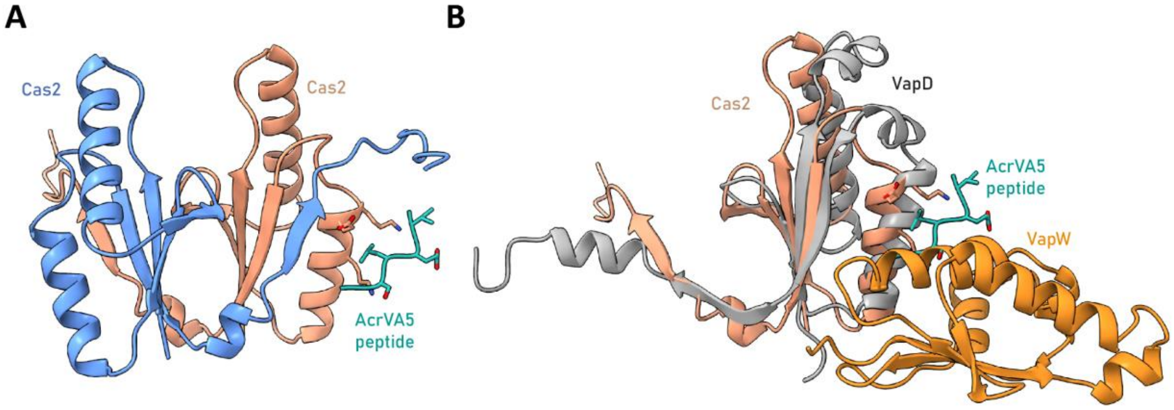

Figure S9. The Cas2 complex with AcrVA5 is similar to that of VapW with VapD A) Structure of Cas2 dimer complexed with a peptide from the anti-CRISPR protein AcrVA5 (PDB ID: 8IA4) **B)** Superimposition of VapD^{Cje}-VapW^{Cje} with Cas2-AcrVA5 peptide complex. Only one chain of Cas2 and VapD are shown.

SUPPLEMENTARY TABLES

Supplementary Table S2. Gene sequences used in this study

No	Gene name	DNA sequence
1	*vapD*^{Mur}	ATGtttgcgatagcatttgacatggtcgtgtcggaccttaaaaagaattatggagagccatacaacaacgcctatttcgagataggtgtaatcttgcgtaaatacgatttctacaatacccaaggtagtgtctatcttaccgagaggaacgatatggcgaatctttacagagcgatagaagccctgaagaaaacgacatggtttcgagattcggtcagagacatccgtgctttcaaggtcgaagactggagcgactttactccagtattcaaggaacagTGA
2	*vapY*^{Mur}	ATGaaagtaggagataaagcgaaagtgtcccctcaactcactggagaatccgactgggtggagggagaggttatagacattgaacagaatccgttcaagggtattgttattgccattaaggacaaagtggggaggatattctttggcgaggagagatatttccagccagttaaactgTAG
3	*vapD*^{Seq}	ATGgtggctaagcaagataactttattcgtttttcgtttaatttcgatttggacatcgctaaattgaaagaatcatacccgtctgataatccaaatggatataagaaagcatggacggatattaaacaatttatggaagcaaatgagtttacacattcacaatattcaggttatgagtccgtaaatgaaatcctttatgctgatgcgtatgcaacgttagaaaagttgcaatccaaatttccttggtttctcaaatgtgctcaagtagctacattgactgaaatcggagaacgctacgatgtcttagaacatctcaatgaacaatcccatcataagtatgatatttcgcatcctgttacgaaatccaaggtttctttattagatgaggtaagttcgatgcgcgatgcatctaaagagttaagtaacgagacgatccttaatcatgatgaactggagcgcTAA
4	*vapW*^{Seq}	ATGcatacatatcaagcaacaccgtggagtttcactatcgagttcgataaaaataaagccgaacagcacggctacgacatcaacgacctctacgactgcgtagacgaaaacgtgcaacgctacggtctgacacgcctcgcacaaggcacatggaaagccaacgaagcagacaaagtagaatcccaatgc

		ctcgccctgtccctgctctccaaacagcaatgggtcatgcacaacatttctatgtttacagtgtgtgaaaaaggtacaacgcctatcgactacgtagagatcgtcaaaaaacacttcccagaacgcgtatatgcaTAA
5	$vapD^{Cje}$	ATGggaatcaatcgcaaggcaatcaactttgatttatctacaaaaagtcttgaaaaatatttcaaagatacacgcgaaccttattctttgattaaaaaatttatgcttgaaaatggtttcgagcatcgccaatattcaggttatacttcaaaagagccaatcaatgaacgccgcgttatccgtatcattaataagctcactaagaaatttacttggcttggcgaatgtgttaaagagtttgatattactgaaatcggagagcagtatagcttaaaagagactatacaagacttatgtgctaaagattttcatcaaaaacttaaagaatttactgaaaaaacaccaaagaatcaaaagttaaaggatctggaacgcTGA
6	$vapW^{Cje}$	ATGgcatacccacttttaggtacacgcatcgtattagatgaagaaaagattttaaaagaaggcaaatataacttagaagatatgtataaaatgatcgatgaatatgctaaagaatcaggaatgatcaaaatcaataaagaaacttatcattgtaaaggggataagtatgatttaggttgtatgactctgtttatctataagtatttgattgattctgaatggtttacgaaaaatgcaaaagaatggatttggattagtgaaaaagaaggaaatagcgatttaatttctgcaagtaaagctgaaggagaaggaatctgggaaTAA
7	$vapD^{Hin}$	ATGtacgcgattgcttttgatttagttgtaaaagacactcaagattatcatccaaagggcgttcaagaggcttatacagacattggcgcagtcttagcaaaatttggttttgttcgcacacaaggaagtttatataccaacatgaatgaagatatggcgaatctctttcaggcaatgaatgcactgaaacagttggcgtggatttctcagtcggtacgcgatattcgtgcttttcgtattgagcaatggtctgattttactgattttattcgtaatTAA
8	$vapD^{Bas}$	ATGtttgccattagctttgatatgtcaatttcagatctcaaaaagcattatggagaaccttataataatgcctactttgagattaaagccatcttacgtaaaaatggctttgaatggattcaaggtagcacatatcttacgcaaagcgaggatttaagtaacctgttccgtgccattgaagcactgaagaaaattgaatggttccgtaagtctgtccgcgatatccgcggttacaaagtagaaaactggtcagactttaccaacattgtaaagaatgaatatgaaTAA
9	$vapD^{Cap1}$	ATGtatgctattgcttttgatatgaatatttcatctttagaaaagcattatgggaaaccatataataatgcttattatgagattgcttcagaacttgaaaaatataacttttaccgcattcaagggagtacctatgtaaccgataataaggatatggggaatttaatgttagccattgatgcccttgcttatattgaatggtttgctaatgctgttcgcgatattcgcgtattccgcattgaggactggagtgatttaactcgtgtagtgaaaattaaatccactaagaaaacatttattaatTAA
10	$vapD^{Cap2}$	ATGcacgctattgcttttgacttaattgtttctgagcttaagaaacattataaagatccatatcacaacgcttatgctgaaattcgtaaagtacttaagcaaaataatttttattggattcaagggagtacttatgctactgaaggcgatttgcgcacccttttcgtgctattcaaaaccttaaaaatatcaaatggttttgcctttcagtacgtgatattcgcgctttcaaaattgaagactactccgattttactcaagaatttcaacttaatatcaatTAA

Supplementary Table S3. Primers used in this study

No	Primer ID	Sequence	Purpose
1	rbs_VapD-Mur_FXba	TTATTATCTAGAGGAGAATCTCTAATGTTTGCGATAGCATTTGACATG	pBAD33_*vapD*^{Mur} vector construction
2	VapD-Mur_RHind	TTATATAAGCTTTTACTGTTCCTTGAATACTGGAGTAAAG	
3	rbs_RelE-ec_FXbaI	TTATTATCTAGAGGAGAATCTCTAATGGCGTATTTTCTGGATTTTGAC	pBAD33_*relE* vector construction
4	RelE-ec_RHindIII	TTATATAAGCTTAGAGAATGCGTTTGACCGC	
5	rbs_CcdB_FXba	TTATTATCTAGAGGAGAATCTCTAATGCAGTTTAAGGTTTACACCTATAAAAGAG	pBAD33_*ccdB* vector construction
6	CcdB_RHind	TTATATAAGCTTATATTCCCCAGAACATCAGGTTAATGG	
7	rbs_VapY_FXba	TTATTATCTAGAGGAGAATCTCTAATGAAAGTAGGAGATAAAGCGAAAGT	pASK_*vapY* vector construction
8	VapY_RHind	TTATATAAGCTTTTACAGTTTAACTGGCTGGAAATATCTCTC	
9	VapD-Mur_FBamHI	TTATTAGGATCCGAAAGTAGGAGATAAAGCGAAAGTGT	pRSF_*hisVapD_strep VapY*^{Mur} vector construction
10	VapD-Mur_RXhoI	TTATATCTCGAGTTACAGTTTAACTGGCTGGAAATATCTC	
11	VapY_FBamHI	TTATTAGGATCCGATGTTTGCGATAGCATTTGACATG	
12	VapY-Seq_ FBamHI	ATTAAAGGATCCGCATACATATCAAGCAACACCGTG	pRSF_*hisVapD_strep VapW*^{Seq} vector construction
13	VapW-Seq_RXho	AATTTACTCGAGTTATGCATATACGCGTTCTGGGAAG	
14	VapW-Seq_FBamHI	ATTAAAGGATCCGGTGGCTAAGCAAGATAACTTTATTCG	
15	VapW-Cje_FBamHI	ATTAAAGGATCCGGCATACCCACTTTTAGGTACAC	pRSF_*hisVapD_strep VapW*^{Cje} vector construction
16	VapW-Cje_RXho	AATTTACTCGAGTTATTCCCAGATTCCTTCTCCTTCAG	
17	VapD-Cje_ FBamHI	ATTAAAGGATCCGGGAATCAATCGCAAGGCAATC	
18	VapD-Mur_F_D7N	ACATGGTCGTGTCGGACCT	D7N *vapD*^{Mur} mutation introduction
19	VapD-Mur _R_D7N	AGGTCCGACACGACCATGTTAAATGCTATCGCAAACAT	

20	pBAD_rev	GATTTAATCTGTATCAGG	Sequencing primers
21	DuetUP1_Primer	GGATCTCGACGCTCTCCCT	
22	DuetUP2_Primer	TTGTACACGGCCGCATAATC	
23	DuetDOWN1_Primer	GATTATGCGGCCGTGTACAA	
24	T7_Ter_Primer	GCTAGTTATTGCTCAGCGG	
25	rbsMazF-mt1_FXbaI	TTATTATCTAGAGGAGAATCTCTAATGAT GCGCCGCGGTG	pBAD33_*mazF*^{mt1} vector construction
26	MazF-mt1_RHindIII	TTATATAAGCTTACGACCATAAGTCGAGA TGCAG	
27	rbsEcoRV_FXbaI	TTATTATCTAGAGGAGAATCTCTAATGAG TCTTCGTTCTGATTTAATTAATGC	pBAD33_*ecoRV* vector construction
28	EcoRV_RHindIII	TTATATAAGCTTATTTTCTTCCTC GGTATATCCAG	
29	rbsVapC-mt20_FXbaI	TTATTATCTAGAGGAGAATCTCTAATGAT CTTCGTCGACACGTC	pBAD33_*vapC*^{mt20} vector construction
30	VapC-mt20_RHindIII	TTATATAAGCTTACTCGGGTCTTACCTCG AC	
35	rbsVapC-mt28_FXbaI	TTATTATCTAGAGGAGAATCTCTAATGAT CGTCGACACGTCG	pBAD33_*vapc*^{mt28} vector construction
36	VapC-mt28_RHindIII	TTATATAAGCTTACCGCCGATCCAGTG	
31	rbsMqsR-ec_FXbaI	TTATTATCTAGAGGAGAATCTCTAATGGA AAAACGCACACCAC	pBAD33_*mqsR* vector construction
32	MqsR-ec_RHindIII	TTATATAAGCTTACTTCTCCTTAAACGAG ACGATC	
33	rbsMenT_FXbaI	TTATTATCTAGAGGAGAATCTCTAATGAC CAAGCCCTATTCGTC	pBAD33_*menT3* vector construction
34	MenT_RHindIII	TTATATAAGCTTATCTTTTCGTCGCCCGA T	

Supplementary Table S4. Data collection and refinement statistics.

**Data collection**	
Beamline	BL32XU
Wavelength (Å)	1.000
Space group	$C222_1$
Unit cell parameters:	
a, b, c (Å)	89.58; 94.81; 137.68
α, β, γ (°)	90; 90; 90
Resolution (Å)	47.40 – 1.95 (2.06 – 1.95)
$CC_{1/2}$	99.5 (52.5)
I/σ	7.67 (0.79)
Completeness (%)	99.9 (99.4)
Unique reflections	83090 (13382)
R_{meas} (%)	20.8 (163.1)
**Refinement statistics**	
Resolution (Å)	47.40 – 1.95 (2.02 - 1.95)
Reflections	83033
R_{work}	0.221
R_{free}	0.249
Number of atoms (total)	3764
Number of protein atoms	3552
Number of water atoms	181
**RMSDs**	
Bond length (Å)	0.007
Bond angles (°)	0.95
**Ramachandran plot**	
Outliers (%)	0.00
Allowed (%)	2.88
Favored (%)	97.12

* Values in the parentheses are for the high-resolution shell

REFERENCES

1. Schneider CA, Rasband WS, Eliceiri KW. 2012. NIH Image to ImageJ: 25 years of image analysis. Nat Methods 9:671-5.
2. Wu D, Piszczek G. 2021. Standard protocol for mass photometry experiments. Eur Biophys J 50:403-409.
3. Daines DA, Jarisch J, Smith AL. 2004. Identification and characterization of a nontypeable Haemophilus influenzae putative toxin-antitoxin locus. BMC Microbiol 4:30.
4. Hu YL, Lee PI, Hsueh PR, Lu CY, Chang LY, Huang LM, Chang TH, Chen JM. 2021. Predominant role of Haemophilus influenzae in the association of conjunctivitis, acute otitis media and acute bacterial paranasal sinusitis in children. Sci Rep 11:11.
5. Kholmina GV, Rebentish BA, Skoblov Iu S, Mironov AA, Iankovskiĭ NK. 1980. [Isolation and characteristics of the new site-specific endonuclease Eco RV]. Dokl Akad Nauk SSSR 253:495-7.
6. Pandey DP, Gerdes K. 2005. Toxin-antitoxin loci are highly abundant in free-living but lost from host-associated prokaryotes. Nucleic Acids Res 33:966-76.
7. Winther K, Tree JJ, Tollervey D, Gerdes K. 2016. VapCs of Mycobacterium tuberculosis cleave RNAs essential for translation. Nucleic Acids Res 44:9860-9871.
8. Cai Y, Usher B, Gutierrez C, Tolcan A, Mansour M, Fineran PC, Condon C, Neyrolles O, Genevaux P, Blower TR. 2020. A nucleotidyltransferase toxin inhibits growth of Mycobacterium tuberculosis through inactivation of tRNA acceptor stems. Sci Adv 6:eabb6651.
9. Christensen SK, Pedersen K, Hansen FG, Gerdes K. 2003. Toxin-antitoxin loci as stress-response-elements: ChpAK/MazF and ChpBK cleave translated RNAs and are counteracted by tmRNA. J Mol Biol 332:809-19.
10. Brown BL, Grigoriu S, Kim Y, Arruda JM, Davenport A, Wood TK, Peti W, Page R. 2009. Three dimensional structure of the MqsR:MqsA complex: a novel TA pair comprised of a toxin homologous to RelE and an antitoxin with unique properties. PLoS Pathog 5:e1000706.
11. Jurėnas D, Chatterjee S, Konijnenberg A, Sobott F, Droogmans L, Garcia-Pino A, Van Melderen L. 2017. AtaT blocks translation initiation by N-acetylation of the initiator tRNA(fMet). Nat Chem Biol 13:640-646.
12. Wilcox B, Osterman I, Serebryakova M, Lukyanov D, Komarova E, Gollan B, Morozova N, Wolf YI, Makarova KS, Helaine S, Sergiev P, Dubiley S, Borukhov S, Severinov K. 2018. Escherichia coli ItaT is a type II toxin that inhibits translation by acetylating isoleucyl-tRNAIle. Nucleic Acids Res 46:7873-7885.
